# Receiving voluntary family planning services has no relationship with the paradoxical situation of high use of contraceptives and abortion in Vietnam: a cross-sectional study

**DOI:** 10.1186/1472-6874-12-14

**Published:** 2012-05-28

**Authors:** Phuong Hong Nguyen, Meiwita P Budiharsana

**Affiliations:** 1 Department of Scientific Research and International Relationship, Thai Nguyen University of Medicine and Pharmacy, Thai Nguyen, Vietnam; 2 Department of Biostatistics and Population, University of Indonesia-Faculty of Public Health, Depok, West Java, Indonesia

**Keywords:** Voluntary family planning (VFP), Contraceptive use, Induced abortion, Counselling, Access, Vietnam

## Abstract

**Background:**

Vietnam shows a paradoxical situation where high contraceptive prevalence goes together with high abortion rates. This study examined the associations between self-reports of having received voluntary family planning (VFP) services and induced abortions.

**Methods:**

A cross sectional survey was conducted in Thai Nguyen province, covering a total of 1281 women. Data were derived from a sample of 935 married women aged 18–49 years who were ever-users (93.5%) and current users of contraceptives (84%), and had completed birth histories. The dependent variables were the likelihood of having an induced abortion and repeated (two or more) induced abortions. The main independent variable was having received the three VFP dimensions (counselling, broader information, and access to availability). The association was examined using multivariate logistic regressions, taking into account women’s socio-demographic characteristics.

**Results:**

The overall induced abortion percentage was 19.4 per 100 pregnancies. None of the three VFP dimensions was significantly associated with the odds of having an induced abortion or having repeated induced abortions. Mother’s age of 35 or older, having more than three living children, and ever used female contraception methods significantly doubled or more the odds of having an induced abortion and significantly tripled the odds of having repeated abortions.

**Conclusions:**

Results indicate that women receiving VFP services were not less likely to have induced abortions. The provision of family planning counselling, information on contraceptive method mix, and management skills to ensure availability, are in need of reinforcement in a new set of policy and program strategies in the future.

## Background

High induced abortion rates (over 1,000,000 abortion per year) and high prevalence of contraceptive use (79%) created a paradoxical situation in Vietnam [1-4]. Around 1999, the country’s family planning services included the three Voluntary Family Planning (VFP) dimensions: family planning counselling; broader information on contraceptive methods that lead to informed voluntary choice; and access to and availability of preferred method(s), all of which encourage contraceptive users to continue using the method and therefore should reduce the need for induced abortions [5]

The family planning program in Vietnam has gone through a long journey. It began as an unofficial program in 1963, was officially instituted through the Council of Ministers Decree No 162-HDBT/1988 and was further reinforced by Resolution 4/1993. The emphasis was solely on a two-child per couple policy with 3 to 5 years of spacing [6]. The target population defined by policy makers in the 1980s consisted entirely of married women. The program did not encourage unmarried women to buy and use either condoms or contraceptive pills. Consequently, there were no family planning services, or even information, for young women who were interested in understanding how to use oral pills and their immediate and long-term side-effects. Women were also uncomfortable buying condoms, because the surrounding society perceived doing so as a sign of a casual approach to sexual relations. This situation explains why there was such a high rate of reliance on traditional methods until now, although women knew that traditional methods are less effective in preventing unwanted pregnancies [7]. Family planning policy makers at that time believed that the intrauterine device (IUD) was the most effective way to meet the official fertility targets of a two-children-per-couple policy with 3 to 5 years of spacing. From the beginning, the family planning program in Vietnam relied heavily on IUDs. Strong campaigns to promote exclusive IUD use were conducted in the 1980s, followed in the mid-1990s by campaigns to promote female (tubal) sterilization [8]. Consequently, for decades, only one modern method, the IUD, predominated in Vietnam’s contraceptive method mix. Other modern methods such as pills, injectables and condoms have been given less attention [9].

During that era, the 6-year pre-service training for medical doctors and 3-year pre-service training for nurses/ midwives/physician assistants did not include academic curricula related to the VFP dimensions. An introductory family planning course of less than 20 hours was given to sixth-year medical students only. However, if a physician or nurse/ midwife/ physician assistant wanted to provide abortion services, they could undergo a 2-month certification course at the provincial reproductive health center after graduation. This certification course was an in-service training with similar course content for physicians and nurses/midwives/physician assistants [10].

Abortion services are legal in Vietnam, if provided through public or qualified private facilities, under a Decree that enabled privatization of the health sector in 1989 [10]. However, it is suspected that many abortion services were delivered without MOH certification because the provider’s qualifications, equipment, and facility sanitation did not meet standard medical requirements 10]. Between 1982 and 1994, induced abortions rose six-fold, leading Vietnam to be a country that has one of the highest abortion rates in the world (with over 1,000,000 abortions per year, or an average of 2.5 induced abortions in a woman’s reproductive life) [10,11]. Each year, approximately 500,000 abortions were reported in the public sector, and another one-third to one-half as many were performed in small health clinics which are suspected of contributing to high women’s death rates [4,10,12]. Limited contraceptive choices, improper use of modern contraceptives and reliance on withdrawal or periodic abstinence contributed to this situation [1,2,13]. Findings from a joint study led by the Ministry of Health/World Health Organization in 1999 concluded that ‘lack of counselling and comprehensive information on available contraceptive method mix’ were associated with the high abortion practices [14]. A multi-donor reproductive health project built a network of in-service trainings (clinical and management) for almost 2,000 health facilities at the national, provincial, district, and commune level in 17 provinces between 1994 and 2009, involving a total budget over 20 million dollars. The three dimensions of VFP – provision of family planning counselling, information on contraceptive method mix, and management skills to ensure availability – were included and adopted in the national standards and guidelines for counselling in regard to family planning and safe abortion [15].

The purpose of this study is to examine the associations between receipt of VFP services and seeking induced abortions and repeated abortions.

## Methods

### Study design and sampling description

A cross-sectional survey was conducted between February and May 2011 in four of the nine districts (which account for 52% of the province’s total population) of Thai Nguyen province in northern Vietnam. These districts have high proportions of indigent and ethnic minority women. A representative sample of married women aged 18–49 years was selected from the 4 districts by a two-stage cluster sampling technique. The first stage of sampling selected 40 communes as primary sampling units, with probability of inclusion proportional to the size of the population in each district. The second stage selected 40 individual respondents in each commune by use of systematic sampling. Respondents were informed about the purpose of the study, and written informed consent was obtained from all respondents. All interviews were conducted in private to ensure confidentiality and privacy. Interviewers were trained to protect confidentiality including the identity of every respondent as well as the secrecy of all recorded information. Interviewers were trained medical doctors undergoing specialization to become obstetricians, with sufficient technical ability to distinguish induced abortions, stillbirths, and miscarriages. Each woman was assigned a respondent code so that her identifying information was known only by the lead investigator; no one else was authorized to see the coded list. All completed questionnaires were stored in locked file cabinets so that only limited research personnel had access to the raw data. This cross-sectional survey received ethical approval from the Institutional Review Board of the Institute of Social and Medicine Studies in Vietnam.

### Operational definitions of variables

The main dependent variables consisted of the probability of ever having induced abortion or ever having repeated (two or more) induced abortions. The structure of the data set did not allow computation of induced abortion ratio by birth order, but allowed the calculation of abortion percentages by mother’s age at the time of an induced abortion. A complete pregnancy history (total pregnancies) of each respondent was computed from the sum of: pregnancies ending in live births and still births (fetal deaths of at least 20 weeks’ gestation), infant deaths (less than or equal to 1 year in age), child deaths (older than 1 year), abortions (medical and surgically induced abortions), and miscarriages (spontaneous abortions regardless of gestation period). Total stated abortions divided by total calculated pregnancies generated the maternal age-specific induced abortion percentages, classified for women 18–24 years, 25–34 years, 35–49 years, and for all ages.

The main independent variable, Voluntary Family Planning (VFP), consisted of three dimensions: ever received family planning counselling; ever received information on contraceptive methods leading to voluntary choice; and perception of access or availability of the selected method(s). Counselling was related to ever receiving family planning counselling from a provider (public or private) during the last family planning visit. Information on family planning method(s) was measured by responses to five yes/no questions related to: receiving advice from a health or population worker; receiving information about a variety of methods; effectiveness of chosen method; side-effects and complications; and knowing what to do when side-effects or complications occurred. Access or availability was measured by responses to three questions related to the respondent’s perceptions about the selected method: its availability; whether it had a reasonable price; and how easy it was to obtain and use. The Cronbach’s alpha coefficient of internal consistency was not used because the individual scale items were set the same.

Other key independent variables:

(1) Combined total family planning ‘ever-users’ and ‘current users’ of contraception that was re-categorized into two binary variables: use of female-only methods (yes = 1 and no = 0); and use of couple methods (yes = 1 and no = 0) [16]. Ever-users were women who said they had used at least one family planning method; and current users were women who said they currently use a family planning method to delay or avoid becoming pregnant. Female-only methods included oral pills, intrauterine devices (IUDs), injectables, implants, and female sterilization. Couple methods included male and female condoms, withdrawal, and periodic abstinence, all of which require a certain degree of support and cooperation from husbands/partners.

(2) Women’s age at interview (categorized into 18–24 years, 25–34 years, and 35–49 years);

(3) Number of living children (alive at the time of the survey, grouped as: 1, 2, and 3+);

(4) Ethnicity (Kinh majority and others: Tay, Nung, Dao, H’mong, Sa Chay, San Diu, and other minority groups);

(5) Wealth index that was constructed based on housing characteristics and household assets: housing quality (roof, wall, floor materials); standard services (water, electricity, type of fuel for cooking, type of toilet); fixed-assets (house, number of rooms, land ownership); durable assets and livestocks [17,18]; The wealth index was constructed using principle components analysis [19] and were then divided into quintiles.

(6) Other control variables such as education, occupation, residence, sex of previous child.

### Statistical analyses

Bivariate analysis was carried out to explore the differentials in induced abortions and ever receiving VFP dimensions, using the chi-square test. Logistic regression was used to re-examine the association between induced abortions as a dependent variable and the VFP dimensions, controlling for socio-demographic characteristics and taking into account design effects resulting from clustering at the level of the primary sampling unit. The results are presented in the form of odds ratios (ORs), with 95% confidence intervals (95% CIs). The analysis was conducted using Stata 11.

## Results

### Sample characteristics

**Figure 1:**
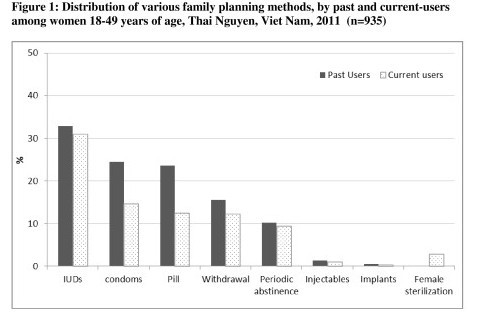
Distribution of various family planning methods, by past and current-users among women 18–49 years of age, Thai Nguyen, Vietnam, 2011 (n = 935).

Of the 1281 women who consented to take part and completed the survey, 935 women were married or living with a partner, were aged 18–49 years, and had completed birth history. High proportions of women were ever-users (93.5%) and current users (84%). Among ever-users, 45.7% had used couple methods and 73.2% had used female methods. Among current users, 43.3% were using couple methods and 56.7% were using female only methods. The distribution of family planning methods by past and current users is shown in Figure 1, indicating that IUDs predominate modern methods, followed by condoms and pills. Other modern methods such as injectables and implants remain less than 1%. Only 14.6% of women stated they had received all three dimensions of VFP. About 43.9% stated they had received counselling, 31.6% had received broader information to make an informed choice, and 37.6% had access to their preferred method.

There were 19.4 induced abortions per 100 pregnancies in the study population. Induced abortion percentage increased with maternal age. The lowest induced abortion percentage (8.8%) was among women aged 18–24 years, which increased to 13.8% among women aged 25–34 years, and reached a peak of 22.2% (about 2.5 times the rate of the youngest group) among women aged 35–49 years.

### Bivariate analyses

Table 1 presents the results of bivariate analysis to explore the differentials in ever having zero, one, or two and more induced abortions and women’s experience in ever receiving VFP (any one, any two or all three dimensions), and other maternal and socio-economic characteristics.

**Table 1:** Bivariate analysis between induced abortions and voluntary family planning dimensions and socio-demographic characteristics

	**Ever had induced abortions (n = 935)**			
Voluntary Family Planning dimensions	**0**	**1**	**≥2**	***P*-value**
Counselling				0.054
No	61.3	25.5	13.2	
Yes	68.8	21.0	10.2	
Information for making informed choice				0.371
No	65.3	22.5	12.2	
Yes	68.8	21.3	9.9	
Access/availability to selected method(s)				0.813
No	64.0	24.2	11.8	
Yes	65.8	22.4	11.9	
Counselling + information				0.083
No	61.6	25.4	13.1	
Yes	68.5	21.2	10.4	
All three components				0.105
No	60.2	27.2	12.7	
Yes	66.9	21.7	11.5	
Age at interview				0.000
18–24	89.9	10.1	0.0	
25–34	77.0	17.8	5.2	
35–49	56.5	26.6	16.9	
Place of residents				0.000
Rural	70.1	21.4	8.5	
Urban	59.7	23.4	16.9	
Ethnicity				0.052
Kinh	68.9	22.8	8.3	
Others (ethnic minorities)	65.3	21.8	13.0	
Education				0.706
Primary and secondary	66.9	22.3	10.8	
High school and higher (college/university)	66.1	21.6	10.3	
Occupation				0.002
Others	60.1	25.9	14.1	
Farmers	69.9	20.1	10.1	
Wealth index (quintiles)				0.001
Lowest	74.3	18.5	7.2	
Second	71.0	21.0	7.9	
Middle	66.5	22.7	10.8	
Fourth	62.5	23.7	13.8	
Highest	57.7	24.4	17.9	
Number of living children				0.000
1	78.7	16.4	4.9	
2	61.6	23.9	14.5	
3+	53.8	29.8	16.4	
Sex of previous child (children)				0.074
Male	63.3	23.9	12.8	
Female	69.3	20.3	10.4	
Ever used contraceptives (couple methods)				
No	70.2	18.9	10.9	0.008
Yes	61.8	25.9	12.3	
Ever used contraceptives (female methods)				
No	76.8	17.9	5.3	0.000
Yes	62.7	23.6	13.7	

The experience of ever having induced abortions did not differ significantly between women who had ever and those who had never received family planning counseling; between women who had ever and those who had never received broader information on contraceptive methods; nor women who had access or those who had no access to their preferred method. Data did not allow an investigation into whether women contraceptive users who received VFP services were more likely to continue using contraceptives, in particular their preferred contraceptive methods. However, the number of women who discontinued the use of contraception was relatively small (95 out of 935).

The probability of having abortions significantly differed by woman’s age at interview, place of residence, type of occupation, wealth, and number of living children; and also in regard to ever-used couple methods and ever-used female methods.

Ethnicity, education, and sex of previous child were not significantly associated with the probability of having induced abortions (one time or repeated).

### Multivariate logistic regression

Table 2 presents results from multivariate logistic regression. None of three VFP dimensions (counseling, information, and access) was shown to be associated with the odds of having an induced abortion or the odds of having repeated abortions.

**Table 2:** Logistic regression on association between voluntary family planning and induced abortion

	**Ever had one abortion (n = 935)**		**Ever had repeated abortion (n = 935)**	
	**OR**	**95% CI**	**OR**	**95% CI**
VFP dimensions				
Counselling				
No	1		1	
Yes	0.74	0.52–1.06	0.64	0.39–1.07
Information for informed Choice				
No	1		1	
Yes	0.97	0.67–1.41	0.98	0.57–1.67
Access/ availability				
No	1		1	
Yes	0.89	0.65–1.20	1.12	0.73–1.74
Age at interview				
18–24	1		1	
25–34	2.37	1.44–3.90		
35–49	3.99	2.35–6.78	2.78	1.54–5.03
Ethnicity				
Others	1		1	
Kinh	0.76	0.55–1.05	1.18	0.72–1.93
Occupation				
Others	1		1	
Farmers	0.57	0.41–0.79	0.64	0.40–1.02
Number of living children				
1	1		1	
2	1.32	0.87–2.01	2.36	1.13–4.95
3+	2.04	1.16–3.61	3.57	1.48–8.57
Sex of previous child				
Male	1		1	
Female	0.72	0.54–0.97	0.62	0.40–0.95
Ever used contraceptives (couple methods)				
No	1		1	
Yes	1.75	1.25–2.46	1.18	0.74–1.90
Ever used contraceptives (female methods)				
No	1		1	
Yes	2.25	1.45–3.40	3.41	1.65–7.08

The odds of having an induced abortion more than doubled with an increase in women’s age (at interview) category: the odds were 2.37 times greater among women aged 25–34, and almost 4 times greater among women aged 35–49 years, compared to women aged 18–24 years. Women aged 18–24 years showed no repeated abortions. But the odds of having repeated abortions (twice or more) among women aged 35–49 years were 2.78 times greater than among women aged 18–34 years (18–24 and 25–34 years pooled).

Ethnicity was not significantly associated with the odds of having an induced abortion nor the odds of having repeated abortions among women of the majority Kinh ethnic group compared to minority women.

Working as a farmer was significantly associated with 43% lower odds of having an induced abortion and 36% lower odds of having repeated abortions. Farmers accounted for 65% of the study’s respondents. In addition, because farmers always need extra hands to help in the field, they did not completely comply with the government’s two-children per couple policy.

Number of living children was significantly associated with a 2.04 fold increase in probability of having an induced abortion. In particular, we found a 3.57 fold increase in probability of having repeated abortions when women already had three or more living children.

Gender of previous child was marginally significant in lowering the odds of ever having induced abortion. Specifically, having previously birthed a daughter resulted in about 28% lower odds of ever having an induced abortion and 38% lower odds of having repeated abortions, compared to women who had previously birthed a son (and were more likely to have reached their desired number of children). The rationale of having previously given birth to a daughter (in a country with two-children per couple policy) means the couple has to have another pregnancy that may give them a son. But if a son has already been achieved, then any subsequent pregnancy may or may not be wanted.

Ever-used couple methods was significantly associated with 75% higher odds of having an induced abortion, but was not significantly associated with increasing the odds of repeated abortions.

Ever-used female methods was significantly associated with 125% higher odds of having an induced abortion, and also 241% higher odds of having repeated abortions.

Logistic regression with current contraceptive users only yielded similar results.

Place of residence, wealth index and education were not included in the final logistic regression model. Place of residence and wealth index were significantly associated with number of induced abortions in the bivariate analyses, but these associations were all cancelled and shown to be no longer associated with either one-time induced abortion or repeated abortions when other independent variables were included in the model.

## Discussion and policy implications

Our study results indicate that none of the three VFP dimensions was significantly associated with the odds of having an induced abortion nor repeated abortions. The proportion of women who ever had one abortion and who ever had more than one abortion did not differ significantly among women receiving counseling, information about modern methods, and access to available methods. These findings are not supportive of the mainstream opinion that receipt of VFP services will influence the use of preferred contraceptives and thus will reduce abortion incidence [20]. However, these findings imply that women in general are still receiving poorly performed family planning counseling and inadequate information/communication about their method of choice, not to mention facing limited contraceptive access/availability.

Accessibility affects nonusers of modern methods and current users of traditional methods in Vietnam [8]. When women who preferred to buy contraceptive pills or condoms instead of IUDs did not receive adequate counseling on how to use oral pills nor adequate information about their immediate and long-term side-effects, they did not feel comfortable buying their preferred methods in the open market and thus they continue to use IUD or chose traditional methods. Figure 1 evidences the fact that current use of IUD is still the predominant modern method in Vietnam. In addition, many Vietnamese family planning providers are still holding the same mindset planted by the IUD campaigns in the 1980s. They did not deliver VFP services seriously, and persisted in treating IUD as the method of attention instead of optimizing access to other modern methods. The attitude of family planning providers certainly will affect future VFP counselling and communication strategies. Policy-wise, increasing the availability of modern contraceptive methods other than IUDs, as well as providing quality information, will increase the use of effective modern family planning methods and decrease the use of traditional methods, leading to change the paradoxical situation of high use of contraceptives and high abortion in Viet Nam.

Performing VFP needs more time than the usual 3–5 minutes of provider-client interaction that is typical at the primary care level. A prerequisite of applying additional services such as VFP is a well-motivated workforce, according to a joint-study conducted by researchers from the Hanoi School of Public Health and the Royal Tropical Institute (KIT) in 2003 [21]. It is noteworthy that the data for the Dieleman et al. 2003 study were collected in two provinces adjacent to Thai Nguyen province (where data for our study were collected). This 2003 motivation study was the first of its kind that looked at rural health workers’ perceptions with respect to job motivation at commune and district health centers in rural areas of northern Vietnam [21]. They found that motivation is influenced by both financial and non-financial incentives (such as appreciation by supervisors, colleagues, and the community). Low salaries, difficult working conditions, and lack of communication skills were noted as discouraging factors. Health workers also tend to perceive supervision as control. In addition, selection for training is seen as opaque and unequal, and performance appraisal is not useful [21]. Although the Ministry of Health in Vietnam prioritizes development of a public health network to provide good quality services including family planning, their findings showed that there were still insufficient qualified and motivated human resources in rural areas.

Motivation, or an individual’s degree of willingness to exert and maintain an effort to achieve certain organizational goals, is a complex concept. It needs supervision, performance appraisal, career development, and appropriate training. Not all trainings are adequate and casual supervision visits achieve little. Apparently, in-service trainings by vertical health programs (such as the voluntary family planning dimensions) do not count as significant credit points in a health worker’s curriculum vitae; in fact, they tend to participate because these trainings pay per diems, so they are perceived merely as an income opportunity.

To some extent, the motivational problem found in performing voluntary family planning dimensions is not exclusive to Vietnam. A recent study (2011) that systematically reviews 80 studies from Africa and Asia and the Pacific on quality and performance of private and public ambulatory health care in low- and middle-income countries (published between 1970 and April 2009) concluded that raising the quality of care is a long-term effort. The government has an important role, but supervision, auditing with feedback and quality training have been found to be an effective combination [22].

In order to strenghthen current VFP services in Vietnam, new performance-based measures should be used to record the number of clients counseled and the number of clients given adequate information about IUD and other modern methods. Monitoring of these new measures should be recorded and reported in order to increase the knowledge and availability of all modern methods, not only IUD. Eventually, this will reduce the number of nonusers of pills and condoms and reduce the number of current users of traditional methods. More in-depth training materials about the three VFP dimensions should be incorporated in the pre-service training curricula for students of medicine, midwifery academics, and nurses. The government’s women’s empowerment program should participate in creating messages that encourage women to obtain information about efficacy of a method before choosing one. This change in orientation should make use of the national standards and guidelines on counselling to protect vo-luntarism in family planning, and will contribute to the government’s efforts to reduce abortion incidence, which will eventually change the paradoxical situation of high use of contraceptives and high abortion in Vietnam.

Our study is limited because it is a cross sectional design; therefore caution must be exercised in the interpretation of the observed associations between induced abortion and VFP measures. In addition, data did not include unmarried women, whose induced abortion behaviors could be different from married women. There was also a possibility that induced abortions among the younger maternal age (18–24) were under-reported because an abortion before the first birth was viewed as disgraceful (implying out-of-wedlock pregnancy). In spite of these limitations, this study is the first one to assess the association between VFP dimensions and induced abortion in Vietnam. Considering that our community-based survey yielded close to a 95% response rate, and the study sample was a representative sample of married women aged 18–49 years, selected using a two-stage cluster sampling technique, our findings can be generalized to the population of women aged 18–49 years in Thai Nguyen province and to the wider region of northern Vietnam with the important policy implication for improving family planning services in the country.

## Conclusion

The findings from this study conclude that receiving VFP was not less likely to have induced abortions or repeated abortion services. In other words, there is no relationship between receiving VFP services and the paradoxical situation of high use of contraceptives and induced abortions in Vietnam. Our findings indicated that although the Ministry of Health has initiated the right intervention with VFP trainings for more than 12 years, the provision of family planning counseling, information on contraceptive method mix, and management skills to ensure availability, are still in need of reinforcement. A new set of policy and program strategies in the future (including indepth training on VFP dimensions, new performance-based measures for VFP, monitoring and supportive supervision) needs to reinforce VFP services that can position voluntarism in family planning as a key performance indicator of quality family planning and safe abortion service.

## Competing interests

None of the authors had financial or non-financial competing interests.

## Authors’ contributions

PHN participated in concept and design, carried out data collection, and performed the statistical analysis of data and revised manuscript. MPB participated in concept and design, drafted the manuscript and framework of analysis. All authors read and approved the final manuscript.

## Pre-publication history

The pre-publication history for this paper can be accessed here:

http://www.biomedcentral.com/1472-6874/12/14/prepub
